# How Effective Are the Protected Areas of the Natura 2000 Network in Halting Biological Invasions? A Case Study in Greece

**DOI:** 10.3390/plants10102113

**Published:** 2021-10-05

**Authors:** Aikaterini Christopoulou, Anastasia Christopoulou, Nikolaos M. Fyllas, Panayiotis G. Dimitrakopoulos, Margarita Arianoutsou

**Affiliations:** 1 Department of Ecology and Systematics, Faculty of Biology, National and Kapodistrian University of Athens, 15784 Athens, Greece; aikatchr@gmail.com; 2 Centre for Research and Conservation of Cultural Heritage, Faculty of Fine Arts, Nicolaus Copernicus University, 87-100 Toruń, Poland; 3 Biodiversity Conservation Laboratory, Department of Environment, University of the Aegean, 81100 Mytilene, Lesbos, Greece; nfyllas@aegean.gr (N.M.F.); pdimi@env.aegean.gr (P.G.D.)

**Keywords:** alien species, climate, European network of protected areas, plant invasions, temporal scale, habitat group

## Abstract

Invasive alien plant species represent an important threat to various protected areas of the world, and this threat expected to be further enhanced due to climate change. This is also the case for the most important network of protected areas in Europe, the Natura 2000 network. In the current study we evaluated the distribution pattern of alien plant taxa across selected continental and insular Natura 2000 sites in Greece and their potential spread 15 years since first being recorded in the field. A total of seventy-three naturalized plant taxa were recorded in the 159 sites under study. At the site level and regardless of the habitat group, the ratio of invaded areas increased between the two monitoring campaigns. An increase in the ratio of invaded plots was also detected for all habitat groups, except for grassland and riparian—wetland habitats. Precipitation during the dry quarter of the year was the factor that mainly controlled the occurrence and spread of alien plant taxa regardless of the site and habitat group. It is reasonable to say that the characterization of an area as protected may not be sufficient without having implemented the proper practices for halting biological invasions.

## 1. Introduction

Natural ecosystems are facing several threats because of human-induced global changes [1]. Climatic and land use changes, invasive alien species (IAS), the loss and fragmentation of natural habitats and over-exploitation of natural resources are the most important direct drivers of biodiversity and ecosystem services losses [2,3,4,5]. Protected areas (PAs) play a key role in biodiversity conservation, sustaining ecosystem services and human welfare [4,6,7,8]. Their role is becoming even more crucial under conditions of global change and environmental degradation [9]. Although the designation and maintenance of PAs represents the most common and most important strategy for biodiversity conservation worldwide [10,11], with a steady increase in both the total area and the numbers of PAs [6,8], the number of inadequately managed sites is increasing as well, mainly due to limited funding [12,13]. Apart from the abovementioned pressures, PAs are also threatened by the effects of tourism, fires, pollution, overgrazing, littering and illegal hunting [8,10,11,14,15,16]. Existing threats may interact with climate change, having a negative synergistic effect on the diversity hosted in the PAs. This seems to be the case particularly for biological invasions, one of the most important threats affecting biodiversity worldwide, with severe ecological, economic and societal impacts [1,17,18,19]. Even though it is nowadays well documented that alien species represent a significant risk to the receiving environments, the impact of non-native species is in several cases difficult to predict and/or to define [20], given the existing diversity in species invasiveness and habitats that are susceptible to invasions [21]. A 30-year re-assessment of biological invasions in different PAs of the world suggested that invasive alien plant species (IAPS) pose the greatest continued threat to PAs [9], whereas, according to the United Nations (UN) Intergovernmental Platform for Biodiversity and Ecosystem Services (IPBES) about one fifth of the Earth’s surface, including the global biodiversity hotspots, are at risk due to biotic invaders [22,23]. Factors affecting the risk of alien invasions have been studied at various scales (e.g., for Europe [4], for the Mediterranean biome [24] and at the country level [25,26]), whereas other studies have focused on the invasion risk for specific habitat types (e.g., sandy shores [27]).

Europe has the largest number of PAs in the world [28,29], with the Natura 2000 network being the most important network of PAs, ranging across the continent. Although most of the aforementioned pressures for PAs in Europe have been systematically evaluated, the presence of alien species within protected areas has been poorly studied and the available data on plant invasions are still rather limited [11,14,30,31,32,33,34,35,36]. The spatial patterns of occurrence of alien plant species across the Natura 2000 network have been studied for Greece [33], whereas Perzanowska et al. [31] have assessed the level of invasion in natural habitat types across the Natura 2000 network in Poland. Although natural ecosystems are more resistant to invasions by IAPS [11,36], there is increasing evidence across the world that PAs are also prone to plant invaders [9,23,37]. The vulnerability of a PA to IAPS depends on various factors, including the identity of the existing habitats [24,36,38], the mean distance from the hydrographic and road network [39], the prevailing climatic conditions [34,40], the land uses and the average human population density in surrounding areas [33,41].

The alien flora of Greece consist of 457 plant taxa [42], among which 396 are neophytes (i.e., taxa introduced and established after the year 1500 AD), whereas the remaining 61 are archaeophytes (i.e., taxa introduced and established before the year 1500 AD), based on the distinction made by Pyšek et al. [43]. Among the alien taxa recorded in Greece, 50 established alien taxa are characterized as invasive, the majority of which (48) are neophytes [42].

The first systematic inventory of alien plant taxa in Greece was launched in 2009 [44,45], and it provided detailed information on their status, chorology, life history traits and habitat preference. One of the most important findings of this work was that natural terrestrial habitats of Greece are much less vulnerable to IAPS than disturbed and human-modified ecosystems, with the exception of riparian habitats. Dimitrakopoulos et al. [33] used field data collected during the first monitoring campaign (1999–2000) for the Natura 2000 terrestrial habitats to investigate the factors determining the spatial patterns of occurrence of alien plant taxa. The authors found that the main factors explaining the variation in the spatial occurrence of alien plant taxa were native plant species richness, topography and hydrography, as well as human population density in the surrounding area [33]. Concerning the impacts of alien species both inside and outside the protected areas for Greece, the available data are still limited and are mainly focused on specific IAPS which have negative impacts on native species and recipient habitats, primarily because of specific traits making the invaders more competitive in regard to local flora [46,47,48]. Competition for available temporal niches [46], reductions in native plant diversity [47] and changes in plant–insect interactions are some of the recorded changes for Greece [48] induced by the presence of IAPS. Even less information is available concerning the possible beneficial impacts that alien plant species may have, such as an increase in global carbon sequestration due to the increased biomass of the recipient habitat, coastal protection from erosion due to sand stabilization, etc. [49].

The Natura 2000 network encompasses Europe’s most important species and habitats. It is the largest coordinated network of PAs in the world, extending across all 27 EU countries, both on land and at sea [50]. PAs of the Natura 2000 network include Sites of Community Importance (SCI) and Special Areas of Conservation (SAC), as defined in the European Commission Habitats Directive (92/43/EEC) aiming to maintain habitats, flora and fauna (other than birds) species at a favorable conservation status, and Special Protection Areas (SPA) following the Council Directive 2009/147/EC on the conservation of wild birds [51].

The aim of the current study is to evaluate the geographical distribution pattern of alien plant taxa across selected Natura 2000 sites in Greece, based on the comparison between the data of the first monitoring field campaign and the most recent data collected during the second monitoring field campaign (2014–2015). The specific objectives of our work were: (i) to identify the most frequently recorded species in the Greek PAs during the last 15 years, (ii) to study the possible spread of alien plant taxa into new sites and/or new habitats 15 years after the first national monitoring campaign, and (iii) to identify the factors that control the occurrence and spread of alien plant taxa in the PAs.

## 2. Results

### 2.1. The Most Frequently Recorded Alien Plant Taxa among PAs During the Last 15 Years and Their Distribution

Seventy-three (73) alien plant taxa were recorded for both time-periods (Table S1). Among the recorded alien taxa, 58 taxa are neophytes, 11 are archaeophytes and four are of unknown status. Fifty-four (54) taxa are established, out of which 34 are characterized as invasive. For period A, 52 alien plant taxa (42 established and eight non-established) were recorded, whereas for period B, 41 alien plant taxa (30 established and six non-established) were recorded, respectively. The 10 taxa present in the highest number of Natura 2000 sites and/or the taxa with the highest number of records in total for the two reference periods are presented in Table 1. With the exception of *Arundo donax*, all of them are neophytes, and excluding *Medicago sativa* subsp. *microcarpa*, all the rest are established and characterized as invasive. The species that was recorded in most of the Natura 2000 sites is *Oxalis pes-caprae*, being present in 41 sites and in 174 plots in total. *Arundo donax* is the second most widespread species, being present in 34 sites and in 110 plots, for both reference periods (Table 1).

**Figure 1:**
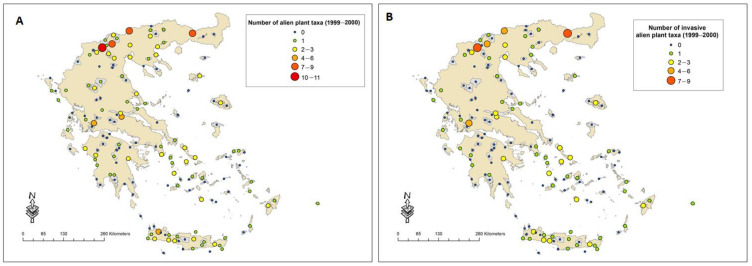
Number of alien plant taxa (**A**) and IAPS (**B**) recorded in each site during period A (1999–2000).

**Figure 2:**
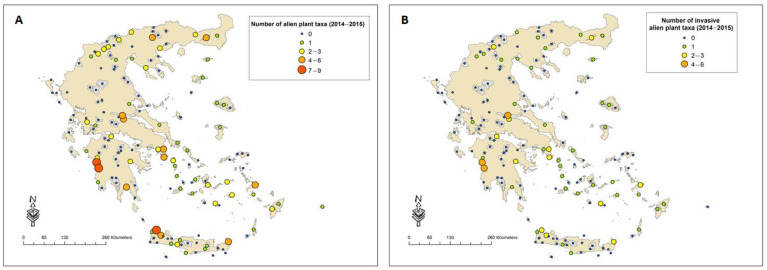
Number of alien plant (**A**) and IAPS (**B**) recorded in each site during period B (2014–2015).

In 53 of the 159 sites studied, no alien plant taxa were recorded in either period. Eighteen sites seem to retain the same number of taxa between the two reference periods, whereas in others the number has increased (33 sites) or decreased (55 sites). During the first reference period, the highest number of alien plant taxa was recorded in wetlands located in Northern Greece (Figure 1A), whereas during the second reference period the highest number of alien plant taxa was recorded in coastal areas in the western Peloponnese and western Crete (Figure 2A). Similar patterns were found for the IAPS (Figure 1B and 2B). Therophytes represent the dominant life form (26% of all taxa recorded), followed by phanerophytes (22%), hemicryptophytes and geophytes (7%). The families with the highest number of alien taxa are Poaceae (16%), Fabaceae (14%) and Asteraceae (12%).

Most alien plant taxa were recorded in habitat types included in the riparian-wetland habitat group (Table S2) for both periods (36 and 30 taxa for 1999–2000 and 2014–2015, respectively). Grassland, coastal and forest habitat groups also hosted relatively high number of alien plant taxa in period A (with 17, 14 and 14 taxa, respectively). The same holds for shrub and forest habitats in period B (with 11 and 9 taxa, respectively). The taxa which occurred in almost all habitat groups were *Oxalis pes-caprae* and *Opuntia ficus-indica* (Table S3).

### 2.2. Spread of Alien Plant Taxa in New Sites and/or New Habitats 15 Years after the First National Monitoring Field Campaign

When considered at the plot level and regardless of the habitat group, the ratio of invaded plots across all Natura 2000 sites under study increased from 3.9% to 6.3% (X^2^ = 54.70, *p* < 0.001) (Table 2). Across all habitat groups, the ratio of invaded plots increased, except for the grassland and the riparian—wetland habitat groups. The percentage of invaded plots increased from 2.2% to 4.2% in forests, with this change identified primarily in the 100–200 m^2^ plot size bin (X^2^ = 7.89, *p* < 0.001). In the shrubland habitat group, the percentage of invaded plots increased from 1.8% to 4.4%, driven mainly by changes in the 20–50 m^2^ bin (X^2^ = 15.25, *p* < 0.001). In the rock habitat group, the ratio increased from 0.8% to 7.0% mainly in the 0–30 m^2^ bin (X^2^ = 31.09, *p* < 0.001). The percentage of invaded plots increased from 1.9% to 6.2% in the coastal habitat group, with this change identified in the 15–30 m^2^ bin (X^2^ = 18.97, *p* < 0.001) (Table 2).

### 2.3. Factors Controlling the Occurrence and Spread of Alien Plant Taxa in the PAs

The mixed-effects model selection procedure revealed that the optimum random structure for modeling delta across sites and habitat groups was the one in which both terms were included in the random component (cAIC = −488.55), suggesting that differences in the percentages of invaded plots varied between sites and habitat groups in a random way. The top-down reduction procedure maintained five terms for the fixed-effects component of the optimal model (no. 4), highlighting a positive effect of Latitude, T_min_ and P_A_ on the delta value and a negative effect of T_A_ and P_dq_ (Table 3).

After identifying significant variations in the delta value between habitat groups, the GLM analysis focused on the factors that control differences in the percentage of invaded plots within each habitat group (Table 4). Within the forest and the shrubland habitat groups, shifts in the invaded areas between the two reference periods were positively related to latitude and negatively to P_dq_. Annual precipitation was also positively related to an increase in invaded areas in the shrubland habitat group. Across the rock habitats, changes in the invaded areas were negatively related to the density of the hydrographic network. Interestingly, insularity had no effect on delta values in any habitat group. The similar differences in the ratio of invaded plots between the two study periods for insular and continental sites is shown in histograms of delta values in the Supplementary Materials (Figure S1).

## 3. Discussion

Seventy-three (73) alien plant taxa were recorded during the two reference periods in the 159 sites of the Natura 2000 network examined in the current study, representing 16% of the total alien flora of Greece [42]. A comparable percentage (< 20%) has been reported for the PAs of the Czech Republic [52]. The natural vegetation of protected areas seems to buffer plant invasion establishment, relative to the comparable non-protected areas [14,53,54]. 

The recorded alien plant taxa were mainly found in Poaceae and Asteraceae, followed by Fabaceae, as has been also reported in other studies, e.g., for Greece [45,55], the Mediterranean biome [24] and worldwide [56]. It is known that these families represent the largest vascular plant families. However, species of these families find important uses in agriculture, so their high representation in the alien flora recorded may also reflect their use in the world economy [25]. Moreover, species belonging to these families have traits permitting them a high level of invasiveness—a high reproductive rate and strong resistance to grazing through metabolic products for Asteraceae [57], increased efficiency dispersal modes in both Poaceae and Fabaceae and characteristic structural units related to grass inflorescence and nitrogen fixation ability in Fabaceae [58].

In terms of life forms, the therophytes were the group with the most representatives, followed by phanerophytes. This pattern has been observed in other regions as well (e.g., [52] for the Czech Republic, [54] for Europe as a whole, [59] for China and [55] for the European Mediterranean Basin). Comparing the life forms of alien and native flora [45,60], there were more alien taxa with phanerophytic life forms and less hemicryptophytic and chamaephytic plants, which was not the case for native flora, of which phanerophytes were the least popular life form. This observation could be related to the fact that the most common introduction pathway for alien plants is to escape from ornamental and horticultural activities [61,62].

From the 18 terrestrial plant species included in the ‘100 of The Worst’ list of DAISIE for alien species in Europe [54,63] and in the more recent list of taxa tagged as ‘high-impact’ in the European Alien Species Information Network (EASIN) dataset [64], six were found within the examined Natura 2000 sites. According to Dimopoulos et al. [42], there are 50 IAPS in Greece, 34 of which were found in protected areas throughout Greece (see Table S3). Out of the four species (*Acacia saligna*, *Ailanthus altissima*, *Ludwigia grandiflora*, *Ludwigia peploides*) of EU Regulation 1143/2014 on the prevention and management of the introduction and spread of invasive alien species which are found in Greece, only two were found to be present in Greek PAs: *Acacia saligna* and *Ailanthus altissima*. Risk assessments for these species in both protected and non-protected areas are needed, whereas special actions for controlling their introduction pathways should also be undertaken [65].

*Oxalis pes-caprae*, a geophyte of African origin [66], being the only species that was present in all six habitat groups, was found in the highest number of sites and exhibited the highest number of records for both reference periods. The ecology of *Oxalis pes-caprae* has been extensively studied in the Mediterranean (e.g., in Spain [67,68] and in Mediterranean islands [66,69]) due to its common presence in urban areas, cultivated areas, olive groves and at the borders of shrublands and forests, where it forms a dense layer that impedes the development of other species, especially during autumn to early spring [45]. Although previous work in Greece [45] reported that it is confined to disturbed areas, it appears now to have invaded natural ecosystems as well, even within protected areas. *Arundo donax*, a species included in the Invasive Species Specialist Group (ISSG) list of 100 worst alien species [70], was recorded in 49 and 69 Natura 2000 sites, respectively, for the two reference periods. It has generally been considered native to sub-tropical Eurasia (including the Mediterranean Basin, Middle East, northern India and East Asia), but the origin of invasive populations remains unknown [71]. That is why the status of the species in Greece remains uncertain, since it is considered native by several authors [72,73], and invasive by others [74].

Riparian—wetland habitats, as well as grasslands, hosted the highest number of alien taxa during the first reference period, without any significant change through time (Table 3). The observed higher number of alien species in riparian-wetland systems relative to other habitats are in accordance with the results from other studies conducted in Greece [44,45,55], Europe [32,36,55] and wider areas throughout the Mediterranean biome [2,24,75]. In the Mediterranean region, water availability in wetlands compared to drier habitats increases their susceptibility to biological invasions [76]. In addition, floods that occur every year in wetlands can transport segments of alien species downstream and thus promote their spread and establishment [77,78,79]. Highly disturbed habitats such as grasslands, with increased proximity to urban areas and transportation networks, increase the propagule pressure of potentially invading species [80], both for continental and insular Natura 2000 sites.

Regarding the factors that may influence the presence and spread of alien plant taxa across all habitats, our results show that climate is the major driver. Warmer and drier sites, with low precipitation during the dry quarter of the year, tend to accumulate a higher number of alien plant taxa across the two reference periods, confirming the findings of Landi et al. [34]. This is also in line with the results of other studies that found more alien species in lower-elevation areas relative to higher ones [33,34,81,82]. Our results support the habitat heterogeneity hypothesis for plant invasions [83,84]. At the habitat level, increased summer water precipitation [85], as well as annual precipitation, e.g., [86], has been found to increase annual tree growth and thus tree stand productivity leading to increased resistance to plant invasions. Limitations of the current study are (a) the data are deficient in terms of variations in the number of native species between time-periods; (b) the possible changes in population size and land use patterns within and around the PAs through time; and (c) the lack of visitation data for PAs. In addition, parameters related to the shape of the protected areas, their connectivity and the degree of disturbances through time (e.g., forest fires, flooding, etc.) are factors that may greatly influence the risk of biological invasions [14,87]. Future research, taking into account the above-mentioned factors, is expected to increase the explanatory power of our models.

Comparison of the spread of alien plant taxa in Natura 2000 sites between the two reference periods indicates that the ratio of invaded areas increased across all Natura 2000 sites. Although Mediterranean islands are considered more vulnerable to IAPS [47] than continental sites, the results of the current study do not show any significant difference in the ratio of invaded plots between continental and insular sites.

Our results show that protected areas are prone to invasion over time, especially if they do not undergo management or undergo no effective management [88], or if specific conservation actions have not taken place (e.g., the implementation of effective legislation). It is thus questionable whether the current status of the Natura 2000 network is sufficient to conserve biodiversity against global-change-related threats. Based on the new legislation recently passed by the Greek government (Law 4685/2020, Government Gazette 92/A/7-5-2020), all Natura 2000 sites fall under the responsibility of the management authorities. Future studies should consider whether this change in status could be sufficient to halt new invasions or the expansion of the current distribution of alien plant taxa.

## 4. Materials and Methods

### 4.1. Study Sites

**Figure 3:**
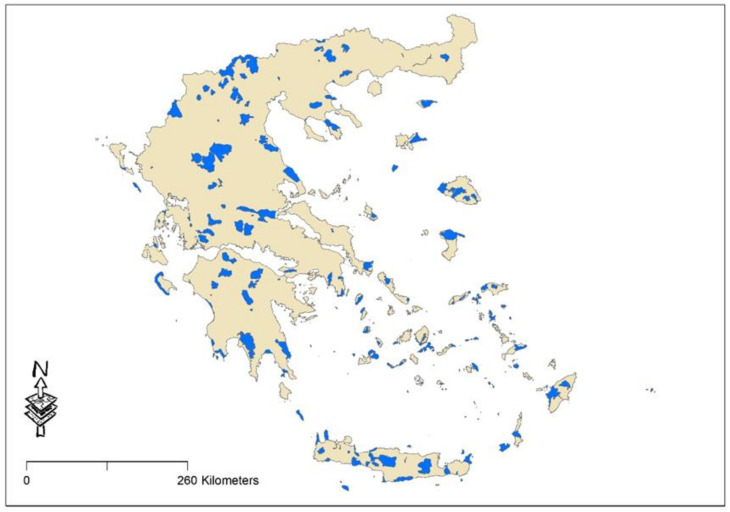
The 159 Natura 2000 sites under study.

The Natura 2000 network in Greece comprises 446 sites, of which 207 are SPAs and 265 are SCIs/SACs [50]. These sites cover 35,982 km^2^ and represent approximately 27% of the total land area of the country [88]. The current study covers 159 continental and insular Natura 2000 sites (denoted as sites) (Figure 3), which are characterized either as SCIs/SACs or as SACs and SPAs. The selected sites are those for which data for the presence and abundance of alien plant species were feasible to derive, covering the years 2000 and 2015 (for details, see next paragraph). The size of the Natura 2000 sites under study varied from less than 1 km^2^ to 618 km^2^. The number of habitat types per Natura site also varied greatly, from 5 to 46 habitats. Mean annual precipitation over those sites varied between 379 to 1734 mm and the mean annual temperature ranged between 4.4 °C and 19.2 °C. Detailed information for each SAC can be accessed through the Natura 2000 Standard Data Forms, available online on the Natura 2000 Network Viewer [50,89]. Within the 159 sites, a total of 9392 plots were sampled during the 1999–2000 period and 9028 plots were sampled during the 2014–2015 period.

### 4.2. Data Preparation

Data on the occurrence of alien plant species were extracted from two databases of the Hellenic Ministry of Environment and Energy. The two databases host data that were collected during the two national monitoring campaigns for the mapping and monitoring of habitat types within the Natura 2000 network. The first monitoring campaign took place during the years 1999–2000 (period A) and the second during 2014–2015 (period B). Sampling was performed using comparable methodologies and efforts in both field campaigns. The number of alien plant taxa was recorded in vegetation sampling plots (relevés), in which data on plant abundance were collected using the transformed (9-point) Braun–Blanquet scale [90] and following the methodology reported in Dimopoulos et al. [91]. The recorded alien plant taxa were classified as established or casual, following [43] and [92]. Taxa of which the status was not clear were classified as unknown.

Each sampling plot was allocated to a Natura 2000 site and one of the following habitat group types: forest, shrubland, coastal, grassland, rock or riparian-wetland (Table S2) as in EUNIS [93] (see [45]). The number of alien plant taxa per habitat group was also estimated for each site.

For each Natura 2000 site, data for average annual temperature (T_A_), minimum annual temperature (T_min_), total annual precipitation (P_A_) and precipitation during the dry quarter (P_dq_) (Table 4) were extracted from the ‘WorldClim-Global Climate Data’ resource [94] at the geographical center of the site, at a spatial resolution of ~1 km^2^. From several variables that can be used as a proxy of socio-economic factors and human-induced drivers of biological invasions, for our analysis we selected the road network, since the density of roads expresses a significant part of the anthropogenic impact in protected areas. Roads have been shown to promote species establishment from the surrounding areas within protected areas, e.g., [41,95], both due to roadside disturbance phenomena and because they act as corridors for propagules transport by vehicles.

The length of the hydrographic and road network within each site was estimated using ArcMap 10.4. The density of the road and hydrographic network was estimated by dividing the length of each network with the total area of the site, as provided in the standard data-entry form (SDF) prepared for inserting field data during the field campaigns. SDF and national mapping of habitat types within the Natura 2000 sites were used to calculate the number of habitat types of each site. The number of native plant taxa within each site was derived from Dimitrakopoulos et al. [33], after revising and updating the dataset for possible nomenclature changes (following [72,73]) and/or re-evaluations of their alien status [42,96]. Information on species life forms and introduction pathways was derived from previous studies [42,45,72,73,96] and the web-based platform “Alien Plants in Greece: A web-based platform [97]”.

### 4.3. Statistical Analysis

To study the potential spread of alien plant taxa between the two reference periods, we counted the number of sampling plots in which at least one alien plant taxon was found and estimated the ratio of “invaded” to total sampling plots. This ratio was estimated at the country level (regardless of site, habitat group and plot area), at the habitat group level (estimating the ratio within each habitat group regardless of site and sampling plot area) and at the habitat group and sampling plot area bin level (after defining area bins for each habitat group and estimating the ratio for each area bin). The area bin increments were discrete for each habitat group in order to account for differences in ecological processes that could affect the number of species found. For example, a finer area bin increment was used for grasslands compared to forests. Differences in the area covered between the two periods were then statistically evaluated by running a proportion test for each of the three grouping levels. All data processing and analysis were carried out using the R statistical language [98] and the tidyverse package [99].

To identify the factors driving the potential changes in the ratio of “invaded” plots between the two field campaigns, we followed a two-step procedure. We initially used a linear mixed-effects model analysis [100] to express the difference between the portion of invaded plots (delta) as a function of the site’s area, latitude, annual average and minimum temperature, total and dry quarter precipitation, hydrographic and road network density and insularity (i.e., whether the site was continental or insular). The predictor variables used are summarized in Table 4 and were standardized before being used in the analysis. To account for the hierarchical structure of the dataset, consisting of plots within sites and plots allocated to a particular habitat group, we fitted four linear models, one with site and habitat group as random effects, one with site as a random effect, one with habitat group as a random effect and another one with no random effect term. We first fitted the beyond-optimal models, where the fixed component contained all explanatory variables, to find the optimal structure of the random component [101], using the restricted maximum likelihood (REML) method. The optimal random structure was identified by comparing the conditional Akaike information criteria (AIC) using the package cAIC4 [102]. After finding the optimal random structure, we followed a top-down approach [101] to identify the optimal fixed structure of the model, sequentially removing the least significant predictors and comparing the nested models (fitted with the maximum likelihood (ML) method), using a likelihood ratio rest (LRT).

As a second step, focusing on the factors that could potentially drive the changes in the ratio of invaded plots within each habitat group, we implemented a generalized linear models (GLM) analysis. Again, the response variable was the difference of the ratio of invaded plots (delta) between the two study periods, and the predictor variables were the ones summarized in Table 5. In particular, latitude and the area of the Natura 2000 sites were used to incorporate biogeographical effects, average annual temperature and total annual precipitation to express the average annual climate of the sites, minimum annual temperature and precipitation during the dry quarter of the year to account for the double stress theory in Mediterranean-type ecosystems [103] related to the species tolerance limits, and road and hydrographic network density to account for potential corridors for alien species dispersal [104,105]. Interactions between predictors were not considered. All GLMs were fitted using a Gaussian error distribution. Initially, a full model including all predictor variables was developed, which was subsequently reduced to the optimum model by dropping each variable and considering reductions in the Akaike information criterion (AIC) [98]. 

## 5. Conclusions

The results of the current study suggest that protected areas without the proper management and conservation practices do not necessary place a barrier on the expansion of alien plant species. Since prevention is the most effective way to combat the risk of biological invasions [106,107], both in terms of minimizing their impacts on biodiversity and ecosystems but also in terms of the economic cost, repeated studies evaluating the presence and distribution of alien plant species, together with proper management, are required. This is of particular importance for the near future, especially since climate change is expected to further increase the risk of biological invasions [36,108], although it might be difficult to predict and generalize how invasive species will interact with climate change at the site level.

## Figures and Tables

**Table 1:** The ten most abundant alien plant taxa, their percentage of records in regard to the number of Natura 2000 sites and the number of plots with the presence of each taxon and the number of different habitat groups where each one has been recorded. More information on invaded habitats is provided in Table S3.

Taxa	Family	Percentage (%) of Natura 2000 Sites with the Presence of Each Taxon			Number of Plots	Number of Habitat Groups
		Period A	Period B	Total		
*Oxalis pes-caprae*	Oxalidaceae	20.8	13.8	25.8	174	6
*Arundo donax*	Poaceae	9.4	15.1	21.4	110	3
*Paspalum distichum*	Poaceae	5.0	6.3	9.4	50	2
*Erigeron canadensis*	Asteraceae	6.3	0	6.3	43	4
*Xanthium spinosum*	Asteraceae	4.4	1.3	5.0	63	3
*Ailanthus altissima*	Simaroubaceae	2.5	3.1	5.7	26	4
*Carpobrotus edulis*	Aizoaceae	0	5.7	5.7	12	2
*Medicago sativa* subsp. *microcarpa*	Fabaceae	5.0	0	5.7	10	4
*Robinia pseudoacacia*	Fabaceae	0.6	4.4	4.4	11	2
*Agave americana*	Asparagaceae	1.3	3.8	5.0	9	4

**Table 2:** Comparison of the number of invaded sampling plots between the two reference periods.

	2000 Campaign			2015 Campaign				
	Presence	All	Ratio	Presence	All	Ratio	X^2^	*p*-Value
Plot level	367	9392	0.039	570	9028	0.063	54.70	<0.001
Habitat group Level	Presence	All	Ratio	Presence	All	Ratio	X^2^	*p*-value
**Forest**								
All plots	60	2688	0.022	109	2608	0.042	15.62	<0.001
0–50	7	111	0.063	1	15	0.067	0.00	1.000
50–100	18	663	0.027	1	21	0.048	0.00	1.000
100–200	23	927	0.025	72	1487	0.048	7.89	<0.001
200–300	5	486	0.010	1	28	0.036	0.10	0.754
300–400	5	281	0.018	34	1053	0.032	1.29	0.255
400+	2	220	0.009	0	4	0.000	0.00	1.000
**Shrubland**								
All plots	46	2543	0.018	81	1835	0.044	24.77	<0.001
0–20	0	113	0.000	7	286	0.024	1.57	0.210
20–50	7	611	0.011	72	1491	0.048	15.25	<0.001
50–100	35	1481	0.024	0	19	0.000	0.00	1.000
100+	4	338	0.012	2	39	0.051	1.41	0.235
**Grassland**								
All plots	37	690	0.054	28	791	0.035	2.50	0.114
0–5	1	69	0.015	10	134	0.075	2.15	0.143
5–10	3	36	0.083	0	3	0.000	0.00	1.000
10–20	3	142	0.021	15	591	0.025	0.00	1.000
20–30	2	178	0.011	0	39	0.000	0.00	1.000
30+	28	265	0.106	3	23	0.130	0.00	0.986
**Rock**								
All plots	6	674	0.008	50	714	0.070	31.90	<0.001
0–30	1	281	0.004	42	589	0.071	17.17	<0.001
30–50	0	155	0.000	8	122	0.066	8.26	0.004
50+	5	238	0.021	0	3	0.000	0.00	1.000
**Coastal**								
All plots	33	1704	0.019	105	1705	0.062	38.03	<0.001
0–15	4	456	0.009	0	33	0.000	0.00	1.000
15–30	9	563	0.016	95	1466	0.065	18.97	<0.001
30–50	15	355	0.042	9	187	0.048	0.01	0.923
50–100	3	283	0.011	1	6	0.167	2.17	0.141
100+	2	47	0.043	0	8	0.000	<0.001	1.000
**Riparian**—**Wetland**								
All plots	185	1060	0.175	196	1351	0.145	3.65	0.056
0–5	10	119	0.084	29	240	0.121	0.77	0.382
5–10	10	45	0.222	1	6	0.167	0.00	1.000
10–20	22	80	0.275	84	416	0.201	1.72	0.190
20–50	53	322	0.165	10	89	0.112	1.09	0.296
50–100	52	237	0.219	2	6	0.333	0.03	0.868
100+	38	257	0.148	70	594	0.118	1.20	0.273

**Table 3:** Top: Results of the model selection procedure for the mixed-effects model of the difference in the invaded plot ratio between the two study periods (delta), showing model structure, the number of random parameters, degrees of freedom (df), log-likelihood (LL), conditional AIC (cAIC) and the difference in cAIC between the various random component models. Bottom: Parameter estimates of the fixed effects of the optimal random structure model (no. 4).

Model No.	Model (beyond Optimal)	Random Parameters	df	LL	cAIC	ΔcAIC
1	only fixed effects	0	11	224.25	−426.49	62.06
2	random intercept for site	1	79.73	320.86	−482.26	6.29
3	random intercept for habitat group	1	14.71	229.85	−430.28	58.27
4	random intercept for site and habitat group	2	84.12	328.49	−488.55	0
	Fixed effects of optimal model (no4)	Estimate	se	df	t	*p*
	intercept	0.021	0.012	11.3	1.823	0.095
	Lat	0.059	0.020	123.6	2.918	0.004
	T_A_	−0.117	0.037	132.8	−3.189	0.002
	T_min_	0.086	0.039	126.5	2.196	0.030
	P_A_	0.025	0.010	140.4	2.537	0.012
	P_dq_	−0.092	0.039	123.4	−2.387	0.019

**Table 4:** Summary of the GLM analysis across each habitat group. Grassland and riparian—wetland habitat groups are not presented separately, since we did not detect significant differences between the two reference periods (see Table 2). Boldface indicates statistically significant values at *a* = 0.05.

	Forest	Shrubland	Rock	Coastal
Intercept	**0.041**	**0.028**	**0.043**	**0.044**
Area				
Latitude (Lat)	**0.093**	**0.056**	0.067	
Average annual temperature (T_a_)	–0.065	–0.069		
Minimum annual temperature (T_min_)			0.071	–0.051
Total annual precipitation (P_A_)		**0.027**	0.035	
Precipitation during the dry quarter (P_dq_)	**–0.148**	**–0.141**		–0.053
Hydrographic network density			**–0.035**	
Road network density				
Null deviance	2.871	1.238	0.956	0.879
Residual deviance	2.618	1.080	0.848	0.843
pseudo R^2^	0.088	0.128	0.113	0.041

**Table 5:** Summary of variables used in the generalized linear models as predictor variables.

Variable Name	Abbreviation	Average	Range	Units or Scale
Area	Area	96	0.32 to 606	km^2^
Latitude	Lat	-	-	degrees
Average annual temperature	T_A_	13.91	5.92 to 19.03	°C
Minimum annual temperature	T_min_	0.35	−8.8 to 8.78	°C
Total annual precipitation	P_A_	669	379 to 1734	mm
Precipitation during the dry quarter	P_dq_	58.33	4 to 146	mm
Hydrographic network density	Hydro	15,910	0 to 379858	m/m^2^
Road network density	Road	23,447	0 to 393675	m/m^2^

## Data Availability

Nomenclature of alien plant taxa follows Dimopoulos et al. [72,73] and the web-based platform “Flora of Greece web: Vascular Plants of Greece—An Annotated Checklist” (http://portal.cybertaxonomy.org/flora-greece/) [109] (accessed date: 11 June 2021), whereas the checklist of alien plant taxa of Greece is available online at the web-based platform “Alien Plants in Greece: A web-based platform” (https://www.alienplants.gr/) [97] (accessed date: 3 May 2021). Information for each SAC can be accessed from the Natura 2000 Standard Data Forms, available online on the Natura 2000 Network Viewer (https://natura2000.eea.europa.eu/) (accessed date: 3 June 2021). The two databases with field data collected during the first (1999–2000) and the second monitoring campaign (2014–2015) for the Natura 2000 terrestrial habitats can be obtained through a special request to the Hellenic Ministry of Environment and Energy.

## Supplementary Materials

The following are available online at https://www.mdpi.com/article/10.3390/plants10102113/s1, Table S1: Alien plant taxa recorded during the last 15 years in the 159 Natura 2000 sites under study. The nomenclature follows Dimopoulos et al. [72,73] and the web-based platform “Flora of Greece web: Vascular Plants of Greece—An Annotated Checklist” [109]. In the Arch/Neo column, archaeophytes are indicated with Arch and neophytes with Neo. In the Invasiveness column, invasive species are indicated with I and non-invasive with N. Information for the three last columns is derived from Dimopoulos et al. [42] and the web-based platform “Alien Plants in Greece: A web-based platform [97]. Table S2: Habitat types (names and codes) included in each habitat group. Table S3: Invasive alien plant species recorded in the Natura 2000 sites under study and invaded habitat groups. Figure S1. Histograms of delta values (difference in the ratio of invaded plots between the two study periods) for insular (light blue) and continental (pink) Natura 2000 sites for all plots and per habitat group.
