# Supplementary material for: How Effective Are the Protected Areas of the Natura 2000 Network in Halting Biological Invasions? A Case Study in Greece
Source: Plants (Basel). 2021 Oct 5;10(10):2113. doi: 10.3390/plants10102113 (PMC8541334; doi:10.3390/plants10102113)
Supplement: Supplementary file 1 [file plants-10-02113-s001.zip › plants-1401726-supplementary.pdf]

Supplementary Material

Table S1. Alien plant taxa recorded during the last 15 years in the 159 Natura 2000 sites under study. Nomenclature follows Dimopoulos et al. [72,73] and the web-based platform "Flora of Greece web: Vascular Plants of Greece An Annotated Checklist"[109]. In column Arch /Neo archaeophytes are indicated with Arch and neophytes with Neo. In column Invasiveness invasive species are indicated with I and non-invasive with N. Information for the three last columns is derived from Dimopoulos et al. [42] and the web-based platform "Alien Plants in Greece: A web-based platform [97].

a/a	Taxon	Family	Status	Arch /Neo	Invasi-veness
1	*Carpobrotus edulis* (L.) N.E. Br.	Aizoaceae	Established	Neo	I
2	*Tetragonia tetragonioides* (Pall.) Kuntze	Aizoaceae	Established	Neo	N
3	*Amaranthus albus* L.	Amaranthaceae	Established	Neo	I
4	*Amaranthus blitoides* S. Watson	Amaranthaceae	Established	Neo	I
5	*Amaranthus hybridus* L.	Amaranthaceae	Established	Neo	I
6	*Amaranthus hypochondriacus* L.	Amaranthaceae	Established	Neo	N
7	*Amaranthus retroflexus* L.	Amaranthaceae	Established	Neo	I
8	*Amaranthus* sp.	Amaranthaceae	-	-	-
9	*Coriandrum sativum* L.	Apiaceae	Established	Arch	N
10	*Daucus carota* subsp. *sativus* (Hoffm.) Arcang.	Apiaceae	Non-established	Neo	N
11	*Zantedeschia aethiopica* (L.) Spreng.	Araceae	Established	Neo	I
12	*Agave americana* L.	Asparagaceae	Established	Neo	I
13	*Hyacinthus orientalis* L.	Asparagaceae	Non-established	Neo	N
14	*Ageratina adenophora* (Spreng.) King & H. Rob.	Asteraceae	Established	Neo	N
15	*Cotula coronopifolia* L.	Asteraceae	Established	Neo	I
16	*Cynara scolymus* L.	Asteraceae	Non-established	Arch	N
17	*Erigeron bonariensis* L.	Asteraceae	Established	Neo	I
18	*Erigeron canadensis* L.	Asteraceae	Established	Neo	I
19	*Erigeron* sp.	Asteraceae	-	-	-
20	*Erigeron sumatrensis* Retz.	Asteraceae	Established	Neo	I
21	*Symphyotrichum squamatum* (Spreng.) G.L. Nesom	Asteraceae	Established	Neo	I
22	*Xanthium spinosum* L.	Asteraceae	Established	Neo	I
23	*Azolla filiculoides* Lam.	Azollaceae	Established	Neo	I
24	*Opuntia ficus-indica* (L.) Mill.	Cactaceae	Established	Neo	I
25	*Opuntia humifusa* (Raf.) Raf.	Cactaceae	Established	Neo	I
26	*Opuntia* sp.	Cactaceae	-	Neo	-
27	*Beta vulgaris* L. subsp. *vulgaris*	Chenopodiaceae	Non-established	Neo	N
28	*Chenopodium giganteum* D. Don	Chenopodiaceae	Established	Neo	N
29	*Dysphania ambrosioides* (L.) Mosyakin & Clemants	Chenopodiaceae	Established	Neo	I

a/a	Taxon	Family	Status	Arch /Neo	Invasi-veness
30	*Dysphania multifida* (L.) Mosyakin & Clemants	Chenopodiaceae	Established	Neo	I
31	*Cuscuta campestris* Yunck.	Convolvulaceae	Established	Neo	I
32	*Elaeagnus angustifolia* L.	Elaeagnaceae	Established	Neo	I
33	*Ricinus communis* L.	Euphorbiaceae	Established	Arch	I
34	*Acacia saligna* (Labill.) Wendl.	Fabaceae	Established	Neo	I
35	*Acacia* sp.	Fabaceae	-	-	-
36	*Amorpha fruticosa* L.	Fabaceae	Established	Neo	N
37	*Caesalpinia gilliesii* (Hook.) D.Dietr.	Fabaceae	Non-established	Neo	N
38	*Gleditsia triacanthos* L.	Fabaceae	Non-established	Neo	N
39	*Lathyrus sativus* L.	Fabaceae	Established	Arch	N
40	*Medicago sativa* subsp. *microcarpa* Urb.	Fabaceae	Non-established	Neo	N
41	*Retama monosperma* (L.) Boiss.	Fabaceae	Established	Neo	N
42	*Robinia pseudoacacia* L.	Fabaceae	Established	Neo	I
43	*Trigonella caerulea* (L.) Ser.	Fabaceae	-	Neo	N
44	*Morus alba* L.	Moraceae	Established	Arch	N
45	*Morus* sp.	Moraceae	-	-	-
46	*Eucalyptus camaldulensis* Dehnh.	Myrtaceae	Established	Neo	N
47	*Oxalis debilis* Kunth	Oxalidaceae	Established	Neo	I
48	*Oxalis pes-caprae* L.	Oxalidaceae	Established	Neo	I
49	*Phytolacca americana* L.	Phytolocaceae	Established	Neo	I
50	*Arundo donax* L.	Poaceae	Established	Arch	N
51	*Bromus rubens* subsp. *kunkelii* (H. Scholz) H. Scholz	Poaceae	Non-established	Neo	N
52	*Echinochloa crus-galli* subsp. *hispidula* (Retz.) Honda	Poaceae	Established	Neo	N
53	*Echinochloa crus-galli* subsp. *spiralis* (Vasinger) Tzvelev	Poaceae	Established	Neo	N
54	*Eleusine indica* (L.) Gaertn.	Poaceae	Established	Neo	I
55	*Hordeum vulgare* subsp. *distichon* (L.) Körn.	Poaceae	Non-established	Arch	N
56	*Paspalum dilatatum* Poir.	Poaceae	Established	Neo	I
57	*Paspalum distichum* L.	Poaceae	Established	Neo	I
58	*Sporobolus indicus* (L.) R.Br.	Poaceae	Established	Neo	N
59	*Stenotaphrum secundatum* (Walter) Kuntze	Poaceae	Established	Neo	N
60	*Triticum aestivum* L.	Poaceae	Non-established	Neo	N
61	*Triticum* sp.	Poaceae	Non-established	Neo	N
62	*Malus domestica* Borkh.	Rosaceae	Non-established	Arch	N
63	*Prunus cerasus* L.	Rosaceae	Established	Arch	N

a/a	Taxon	Family	Status	Arch /Neo	Invasi-veness
64	*Prunus dulcis* (Mill.) D.A.Webb	Rosaceae	Established	Arch	N
65	*Populus* x *canadensis* Moench	Salicaceae	Non-established	Neo	N
66	*Cymbalaria muralis* G. Gaertn., B. Mey. & Scherb.	Scrophulariaceae	Established	Arch	I
67	*Ailanthus altissima* (Mill.) Swingle	Simaroubaceae	Established	Neo	I
68	*Datura inoxia* P. Mill.	Solanaceae	Established	Neo	N
69	*Datura stramonium* L.	Solanaceae	Established	Neo	I
70	*Nicotiana glauca* Graham	Solanaceae	Established	Neo	I
71	*Solanum elaeagnifolium* Cav.	Solanaceae	Established	Neo	I
72	*Tamarix arborea* B.R. Baum	Tamaricaceae	Established	Neo	N
73	*Tropaeolum majus* L.	Tropaeolaceae	Established	Neo	N

Table S2. Habitat types (names and codes) included in each habitat group.

Habitat group	Natura 2000 code	Habitat type name
Riparian - wetland	3130	Oligotrophic to mesotrophic standing waters with vegetation of the *Littorelletea uniflorae* and/or Isoeto Nanojuncetea
	3150	Natural eutrophic lakes with *Magnopotamion* or *Hydrocharition* - type vegetation
	3170	*Mediterranean temporary ponds
	3240	Alpine rivers and their ligneous vegetation with *Salix elaeagnos*
	3250	Constantly flowing Mediterranean rivers with *Glaucium flavum*
	3280	Constantly flowing Mediterranean rivers with *PaspaloAgrostidion* species and hanging curtains of *Salix* and *Populus alba*
	3290	Intermittently flowing Mediterranean rivers of the *Paspalo-Agrostidion*
	72A0	Reed thickets
	72B0	Societies high reeds
	7210	*Calcareous fens with *Cladium mariscus* and species of the *Caricion davallianae*
	91E0	*Alluvial forests with *Alnus glutinosa* and *Fraxinus excelsior* (*Alno-Padion, Alnion incanae, Salicion albae*)
	91F0	Riparian mixed forests of *Quercus robur, Ulmus laevis* and *Ulmus minor, Fraxinus excelsior* or *Fraxinus angustifolia*, along the great rivers (*Ulmenion minoris*)
	92A0	*Salix alba* and *Populus alba* galleries
	92C0	*Platanus orientalis* and *Liquidambar orientalis* woods (*Plantanion orientalis*)
	92D0	Southern riparian galleries and thickets (*NerioTamaricetea* and *Securinegion tinctoriae*)
Coastal	1210	Annual vegetation of drift lines
	1240	Vegetated sea cliffs of the Mediterranean coasts with endemic *Limonium* spp.
	1410	Mediterranean salt meadows (*Juncetalia maritimi*)
	1420	Mediterranean and thermo-Atlantic halophilous scrubs (*Sarcocornetea fruticosi*)
	2110	Embryonic shifting dunes
	2120	Shifting dunes along the shoreline with *Ammophila arenaria* (white dunes)
	2190	Humid dune slacks
	2220	Dunes with *Euphorbia terracina*
	2230	*Malcolmietalia* dune grasslands
	2250	*Coastal dunes with *Juniperus* spp.
	2260	*Cisto-Lavenduletalia* dune sclerophyllous scrubs
	2270	*Wooded dunes with *Pinus pinea* and/or *Pinus pinaster*
Forest	9130	*Asperulo-Fagetum* beech forests
	9250	*Quercus trojana* woods
	9260	*Castanea sativa* woods
	9280	*Quercus frainetto* woods

Habitat group	Natura 2000 code	Habitat type name
	9290	*Cupressus* forests (*Acero-Cupression*)
	9310	Aegean *Quercus brachyphylla* forests
	9320	*Olea* and *Ceratonia* forests
	9350	*Quercus macrolepis* forests
	9370	*Palm groves of *Phoenix*
	9540	Mediterranean pine forests with endemic *Mesogean* pines
	91M0	Pannonian-Balkanic turkey oak- sessile oak forests
	925A	*Ostrya, Carpinus* and mixed thermophilous forests
	934A	Greek *Quercus coccifera* woods
Grassland	6220	*Pseudo-steppe with grasses and annuals of the *Thero-Brachypodietea*
	6290	Mediterranean subnitrophilous grasslands
	6420	Mediterranean tall humid herb grasslands of the *Molinio-Holoschoenion*
	62A0	Eastern sub-mediteranean dry grasslands (*Scorzoneratalia villosae*)
Rock	8140	Eastern Mediterranean screes
	8210	Calcareous rocky slopes with chasmophytic vegetation
	8220	Siliceous rocky slopes with chasmophytic vegetation
Shrubland	4090	Endemic oro-Mediterranean heaths with gorse
	5160	South-eastern submediterranean deciduous thickets (*Prunion fruticosae*)
	5210	Arborescent matorral with *Juniperus* spp.
	5330	Thermo-Mediterranean and pre-desert scrub
	5340	Garrigues of Eastern Mediterranean
	5350	Pseudomaquis
	5420	*Sarcopoterium spinosum* phryganas

Table S3. Invasive alien plant species recorded in the Natura 2000 sites under study and invaded habitat groups.

Species	Number of Natura 2000 sites with presence of each taxon	Invaded habitat groups
Carpobrotus edulis	9	coastal, riparian
Amaranthus albus	3	grasslands, riparian, shrublands
Amaranthus blitoides	3	coastal, riparian
Amaranthus hybridus	3	coastal, riparian
Amaranthus retroflexus	4	forests, riparian
Zantedeschia aethiopica	1	grasslands, riparian
Agave americana	8	coastal, forests, riparian, shrublands
Cotula coronopifolia	1	coastal, riparian
Erigeron bonariensis	6	forests, grasslands, riparian, shrublands
Erigeron canadensis	10	coastal, grasslands, riparian, shrublands
Erigeron sumatrensis	6	coastal, grasslands, riparian
Symphyotrichum squamatum	5	coastal, forests, grasslands, riparian
Xanthium spinosum	8	coastal, grasslands, riparian
Opuntia ficus-indica	5	coastal, forests, riparian, rocky, shrublands
Opuntia humifusa	1	rocky

**Species**	**Number of Natura 2000 sites with presence of each taxon**	**Invaded habitat groups**
Dysphania ambrosioides	4	coastal, grasslands, riparian
Dysphania multifida	1	grasslands
Cuscuta campestris	1	grasslands, riparian
Elaeagnus angustifolia	1	riparian
Ricinus communis	1	shrublands
Acacia saligna	1	coastal, riparian
Robinia pseudoacacia	7	forests, riparian
Oxalis debilis	1	coastal
Oxalis pes-caprae	41	coastal, forests, grasslands, riparian, rocky, shrublands
Phytolacca americana	2	riparian
Cymbalaria muralis	4	forests, riparian, rocky
Eleusine indica	1	riparian
Paspalum dilatatum	1	riparian
Paspalum distichum	15	grasslands, riparian
Azolla filiculoides	1	riparian
Ailanthus altissima	9	forests, riparian, rocky, shrublands
Datura stramonium	1	riparian
Nicotiana glauca	2	riparian, shrublands
Solanum elaeagnifolium	3	coastal

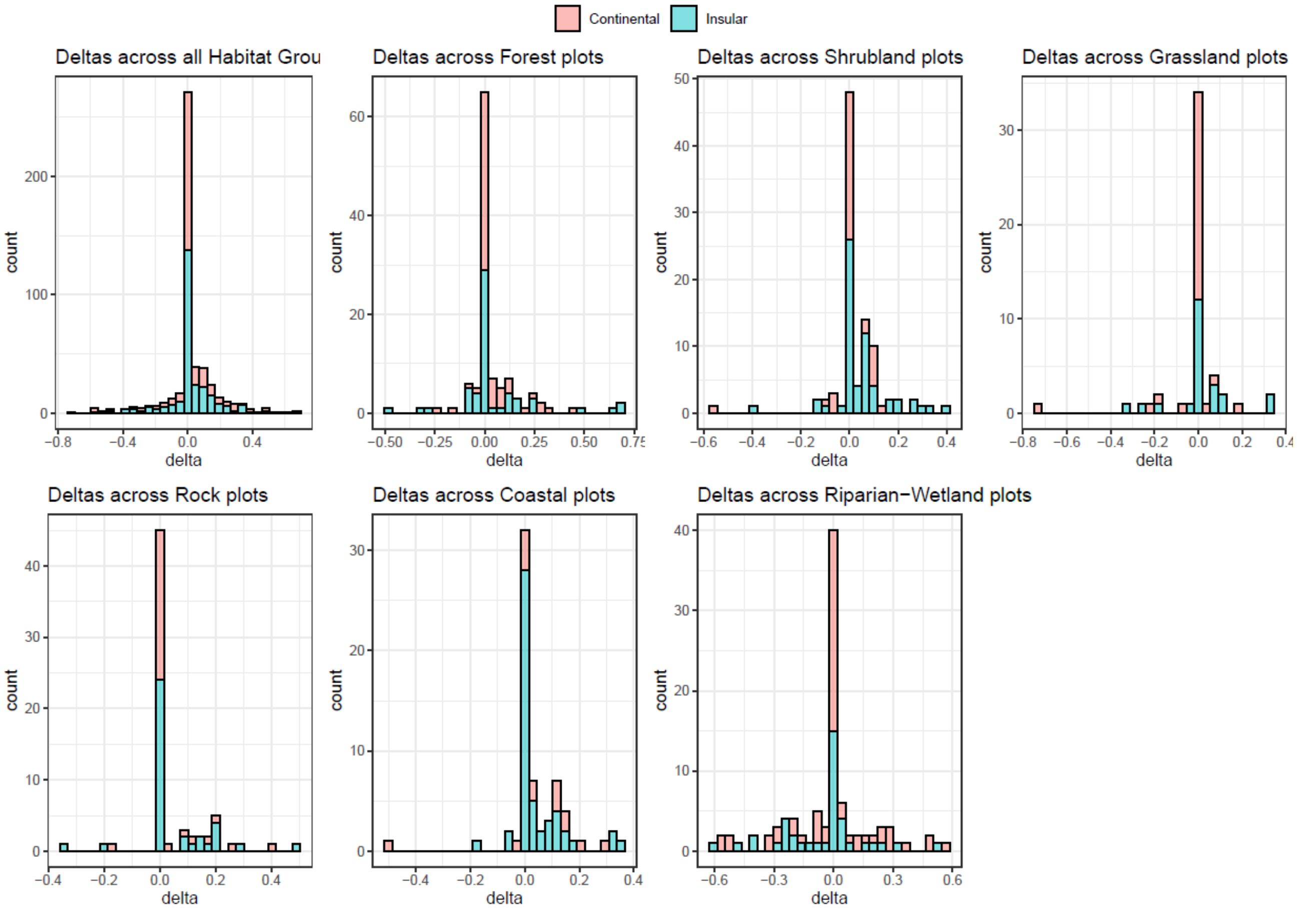

Figure S1. Histograms of delta (difference in the ratio of invaded plots between the two study periods) for insular (light blue) and continental (pink) Natura 2000 sites for all plots and per habitat group.
